# Parent chronic pain and mental health symptoms impact responses to children’s pain

**DOI:** 10.1080/24740527.2018.1518119

**Published:** 2018-10-08

**Authors:** Lauren M. Fussner, Cathleen Schild, Amy Lewandowski Holley, Anna C. Wilson

**Affiliations:** a Division of Behavioral Medicine & Clinical Psychology, Cincinnati Children’s Hospital Medical Center, Cincinnati, Ohio, USA; b Psychology Department, Pacific University, Forest Grove, Oregon, USA; c School of Medicine, Department of Pediatrics, Institute on Development and Disability, Oregon Health & Science University, Portland, Oregon, USA

**Keywords:** parenting, chronic pain, mental health, catastrophizing, protectiveness

## Abstract

**Background:**

Chronic pain is a prevalent health condition associated with parenting difficulties. Pain-specific parenting, such as protectiveness and catastrophizing, may contribute to chronic pain in children. Additional work is needed to test predictors of pain-specific parenting.

**Aim:**

The current study tested parent mental health symptoms as predictors of protectiveness and catastrophizing about child pain and whether comorbid pain and mental health symptoms exacerbate risk for problematic responses to children’s pain.

**Methods:**

Parents with chronic pain (*n* = 62) and parents without chronic pain (*n* = 80) completed self-report questionnaires assessing pain characteristics, mental health symptoms, and pain-specific parenting responses.

**Results:**

Results indicated significantly higher rates of depression, anxiety, and somatization in parents with chronic pain. Depression predicted protectiveness and catastrophizing over and above chronic pain status. Chronic pain status moderated the association between increased anxiety and greater catastrophizing about child pain.

**Conclusions:**

Findings highlight the potential impact of mental health symptoms on pain-specific parenting even when accounting for chronic pain status.

## RÉSUMÉ

**Contexte**: La douleur chronique est un problème de santé courant associé à certaines difficultés en matière de pratiques parentales. Les pratiques parentales spécifiques à la douleur, telles que la protection et la dramatisation, peuvent contribuer à la douleur chronique chez l’enfant. Des études additionnelles sont nécessaires afin de tester les prédicteurs des pratiques parentales spécifiques à la douleur.

**But**: La présente étude cherchait à savoir si les symptômes de troubles mentaux étaient de bons prédicteurs d’une attitude protectrice et de la dramatisation de la douleur chez l’enfant et si la comorbidité de la douleur et des symptômes de troubles mentaux exacerbait le risque de réponses problématiques à la douleur de l’enfant.

**Méthodes**: Des parents souffrant de douleur chronique (n = 62) et des parents sans douleur chronique (n = 80) ont répondu à un questionnaire d’autoévaluation portant sur les caractéristiques de la douleur, les symptômes de troubles mentaux et les réponses parentales à la douleur.

**Résultats**: Les résultats ont indiqué des taux de dépression, d’anxiété et de somatisation plus élevés chez les parents souffrant de douleur chronique. La dépression était un prédicteur de la protection et de la dramatisation en plus de l’état de douleur chronique. L’état de douleur chronique modérait l’association entre l’augmentation de l’anxiété et une plus grande dramatisation de la douleur chez l’enfant.

**Conclusions**: Les résultats mettent en lumière l’effet potentiel des symptômes de troubles mentaux sur les pratiques parentales spécifiques à la douleur, même lorsque l’on tient compte de l’état de douleur chronique.

## Introduction

Chronic pain is one of the most prevalent health conditions, affecting up to 30% of adults.^1,^^2^ Similar to other chronic illness conditions, chronic pain is associated with significant disruption in social, emotional, and physical functioning.^3^ Family functioning is an important social domain commonly influenced by chronic pain. Individuals with chronic pain experience increased family conflict,^4^ decreased family cohesion,^5^ and greater difficulty completing parenting tasks.^6^ The majority of research testing the impact of parent chronic pain on family functioning has assessed parenting and family factors broadly^7^ despite recent work demonstrating the importance of pain-specific parenting factors. Specifically, pain-specific parenting (or problematic pain behaviors) may be a critical mechanism contributing to the transmission of chronic pain from parents to offspring.^8–11^ Importantly, risk for chronic pain is complex and multifaceted.^11^ Additional work is needed to test predictors of pain-specific parenting to increase understanding of factors that place children of parents with chronic pain at greatest risk. Identifying these factors can ultimately aid in the development of targeted preventive interventions that can support parents with chronic pain conditions.

Parent responses to children’s pain behavior (e.g., protectiveness, catastrophizing) may be particularly impacted by parent chronic pain. *Parent protectiveness* refers to behavioral responses (e.g., bringing child special treats) to children’s somatic complaints or pain symptoms, and *catastrophizing* refers to parental cognitions (e.g., ruminating or feeling overwhelmed) about child pain. In a subsample of parents assessed in the present study, we found associations between parent chronic pain and pain-specific parenting responses, such that parents with chronic pain engaged in greater (i.e., more problematic) protectiveness and catastrophizing of children’s pain behavior, relative to parents without chronic pain.^12^ Additional research supports this finding,^13^ suggesting that parents with chronic pain engage in higher levels of problematic pain-specific parenting responses. However, not all parents with chronic pain engage in higher levels of these parenting responses. The current study extends previous findings and tests predictors of pain-specific parenting responses above and beyond chronic pain status.

Mental health symptoms impact a multitude of cognitive and behavioral responses, including thinking patterns, energy level, attention, and sleep.^14,15^ As such, parent mental health has been linked to disrupted parenting.^16,17^ For example, mothers with depression display increased withdrawal and inconsistent discipline^18^ and parents with anxiety demonstrate higher rates of criticism.^19,20^ Theoretical work suggests an association between mental health symptoms and increased attention to physical complaints.^11^ Parent mental health symptoms (i.e., depression, anxiety, somatization) may contribute to pain-specific parenting responses. However, this association has not been assessed, despite high comorbidity rates between chronic pain and mental health symptoms. Specifically, prevalence rates of mood disorders (i.e., anxiety, depression) increase fourfold among adults with chronic pain.^21^ Moreover, given the demands of mental health symptoms and chronic pain, the presence of both conditions (i.e., comorbid mental health and chronic pain) may exacerbate risk for problematic behavioral and cognitive responses (i.e., protectiveness and catastrophizing) to children’s pain. Importantly, mental health symptoms are influenced by a host of social and environmental factors, including the presence of *child* pain. Specifically, parents of children with chronic pain experience higher levels of depression and anxiety compared to parents of healthy youth.^22^ The current study tested parent mental health symptoms in relation to pain-specific parenting, though this relation is likely influenced by child health as well.

Additional research is needed to identify factors that contribute to pain-specific parenting and place children of parents with chronic pain at greatest risk for negative outcomes. The current study begins to address this gap by testing predictors of problematic pain-specific parenting responses across two aims. First, we tested parent mental health symptoms in relation to pain-specific parenting. We predicted that greater mental health symptoms (i.e., anxiety, depression, somatic symptoms) would be associated with higher levels of parent protectiveness and catastrophizing about child pain. Second, we tested whether comorbid pain and mental health symptoms exacerbated risk for problematic pain-specific parenting. We hypothesized that parents with elevated mental health symptoms and current chronic pain would engage in greater protectiveness and catastrophizing behavior than those without chronic pain.

## Method

### Procedure and participants

Study procedures were approved by the institutional review board at the university medical center where data were collected. Data were derived from a longitudinal investigation assessing intergenerational risk for pain. Participants were recruited from a major metropolitan area in the Pacific Northwest region of the United States. Parents without chronic pain (*n* = 80) were recruited from the community; parents with chronic pain (*n* = 62) were recruited via direct mailings and posting of study fliers in specialty medical care clinics for pain, as well as through sharing study information via patient group email listservs. Interested participants contacted the study team and were screened for eligibility criteria. All participants were required to have a biological child between the ages of 11 to 15. Parents with chronic pain were required to have pain for 3 months or longer and currently be receiving specialty medical care for their pain. Parents attended an in-person laboratory visit and provided written informed consent. Self-report measures assessing mental health symptoms and pain-specific parenting behaviors were completed via research electronic data capture REDCap,^23^ a secure computerized survey system used to collect and manage study data. Parents were compensated for their time with a gift card to a local store.

Participants were 142 biological parents (126 mothers, age range 27–59, M = 43.37, SD = 6.54) of children between 11 and 15 years (84 girls, M = 13.03, SD = 1.37). The majority of parents were female and identified their race as Caucasian (87%), with 3.4% identifying as black, 3% identifying as Hispanic, 2.8% identifying as Asian, 2.8% identifying as biracial, 1.4% as American Indian or Alaska Native, and 1.4% as other. Twenty-eight percent of parents completed a bachelor’s degree and annual income ranged from ≤$25,000 to ≥$200,000 with the mean parent income between $50,000 and $74,999.

### Measures

#### Sociodemographics and pain characteristics

Parents self-reported their age, sex, race, ethnicity, income, and education as well as their child’s age and sex. Parents also reported their primary pain problem on a pain problem checklist and on pain characteristics over the past 7 days via the Brief Pain Inventory (BPI^24^).

#### Mental health symptoms

The Brief Symptoms Inventory (BSI-18^25^; was used to assess parental symptoms of depression, anxiety, and somatic complaints. Items are divided equally to yield three subscales assessing separate symptom profiles as well as a total symptom score. Items are scored on a five-point Likert scale ranging from *not at all* (0) to *extremely* (4) and assess the frequency of symptoms over the past week. Subscale scores were calculated as the sum of six subscale items and higher scores indicate greater mental health symptoms. The BSI-18 demonstrates adequate internal consistency across subscales (current sample: α = 0.90 for depression; α = 0.72 for anxiety; α = 0.84 for somatization). Nineteen percent of males and 30% of females scored above the clinical cut point (male score ≥ 10; female score ≥ 13) for overall distress.

#### Parent protectiveness

The 13-item Protect subscale of the Adult Responses to Children’s Symptoms^26,27^ was administered to assess parents’ protective behaviors. *Protectiveness* refers to attentive and solicitous behaviors (e.g., allowing child to miss school/activities, bringing child special treats) in response to children’s pain. Parents are presented with the prompt, “When your child has aches or pains, how often do you …” and respond to each item with the frequency of protective behavior. Items are scored on a 5-point Likert scale ranging from *never* (0) to *always* (4). Items are summed to create a total protect score and higher scores indicate greater protectiveness. The protect subscale has demonstrated sufficient internal consistency (current sample: α = 0.89).

#### Parent catastrophizing

The 13-item Pain Catastrophizing Scale for Parents (PCS-P^28^) assessed the frequency of catastrophizing thoughts and feelings that parents may have in response to children’s pain. Catastrophizing refers to persistent and worrisome thoughts highly focused on children’s pain (e.g., thinking that child’s pain will never go away or get better). The PCS-P is adapted from the Pain Catastrophizing Scale^29^ and the Pain Catastrophizing Scale for Children (PCS-C^30^). Parents respond to items with the stem, “When my child is in pain.” Items are scored on a 5-point Likert scale ranging from *not at all* (0) to *extremely* (4). The PCS-P yields a total summed score ranging from 0 to 52 and three subscales assessing rumination, magnification, and helplessness. Higher scores indicate greater catastrophizing behavior. The total score was used in the current study. Internal consistency estimates in the standardization sample and the current sample (α = 0.89) are acceptable.

## Results

Preliminary analyses were conducted to test distributional assumptions and correlations between study variables. Five participants did not have complete data. A total of 1.14% of values were missing. No differences emerged between participants with complete data and participants with incomplete data (all *P* values > 0.11). Little’s Missing Completely at Random (MCAR) test was nonsignificant (χ^2^ = 8.82, *P* = 0.99), suggesting that data were likely missing completely at random. Missing data were imputed using multiple imputation with 20 imputations. Pooled estimates are shown for all analyses below.^31^ Self-report variables were within the expected range and normally distributed.

Sample characteristics and differences between parents with and without chronic pain are reported in Table 1. Independent samples *t*-tests and chi-square analyses demonstrated significant differences between groups on parent age, education, income, and BPI average pain intensity scores. In the chronic pain group, primary pain problem included fibromyalgia (40.3%), back pain (19.4%), other musculoskeletal pain (e.g., neck pain, shoulder pain; 25.8%), headache (8.1%), and abdominal pain (6.5%). Similar to what was previously reported from a subset of this sample,^12^ parents with chronic pain reported significantly higher levels of catastrophizing compared to parents without chronic pain. Group differences in protectiveness emerged at the trend level (*P* = 0.06), with parents with chronic pain reporting more protective behaviors. Parents with chronic pain were significantly higher on depression, anxiety, and somatization, as measured by the BSI-18. Bivariate correlations showed significant associations between mental health symptoms and protectiveness (depression *r* = 0.29, anxiety *r* = 0.19, somatization *r* = 0.21), as well as mental health symptoms and catastrophizing (depression *r* = 0.47, anxiety *r* = 0.40, somatization *r* = 0.33).

**Table 1.** Sample descriptive statistics and differences between parents with and without pain (*n* = 142).

	Pain (*n* = 62) M (SD)/*N* (%)	No pain (*n* = 80) M (SD)/*N* (%)	*t* (df)/χ^2^	*P*	*d/φ*
Parent sex			0.28 (1)	0.598	0.04
Female	56 (90)	70 (87.5)			
Male	6 (10)	10 (12.5)			
Parent age (years)	42.20 (6.86)	44.61 (6.72)	2.09 (140)	**0.038**	**0.35**
Parent race			1.52 (6)	0.958	0.10
Asian	1 (1.6)	3 (3.8)			
American Indian	1 (1.6)	1 (1.3)			
African American	2 (3.2)	3 (3.8)			
White	55 (88.7)	69 (86.3)			
More than one race	2 (3.2)	2 (2.5)			
Other	1 (1.6)	1 (1.3)			
Not reported	0 (0)	1 (1.3)			
Parent education			22.63 (6)	**0.001**	**0.40**
Some high school	1 (1.6)	0 (0)			
High school/GED	9 (14.5)	6 (7.5)			
Technical school	6 (9.7)	1 (1.3)			
Some college/associate’s	24 (38.7)	15 (18.8)			
Bachelor’s	12 (19.4)	24 (30)			
Master’s or doctoral	10 (16.1)	34 (42.6)			
Income ($)			18.64 (7)	**0.009**	**0.36**
≤25,000	16 (25.8)	4 (5)			
25,000–49,000	18 (29)	16 (20)			
50,000–74,999	9 (14.5)	16 (20)			
75,000–99,999	9 (14.5)	20 (25)			
100,000–149,999	6 (9.7)	11 (13.8)			
150,000–199,999	3 (4.8)	7 (8.8)			
≥200,000	1 (1.6)	6 (7.5)			
BPI Average Intensity	4.34 (1.86)	0.55 (1.15)	−14.03 (93.78)	**<0.001**	**2.46**
BSI Depression	5.83 (5.92)	0.79 (1.58)	−6.52 (67.74)	**<0.001**	**1.16**
BSI Anxiety	5.45 (4.85)	0.97 (1.63)	−6.99 (71.72)	**<0.001**	**1.24**
BSI Somatization	7.29 (4.67)	0.50 (1.12)	−11.20 (66.50)	**<0.001**	**2.00**
Protectiveness	19.90 (9.93)	16.95 (8.81)	−1.87 (140)	0.063	0.31
Catastrophizing	12.49 (8.46)	8.06 (4.92)	−3.67 (92.27)	**<0.001**	**0.64**

BPI = Brief Pain Inventory; BSI = Brief Symptoms Inventory.

Values in bold indicate significant differences between groups.

### Associations among parent mental health symptoms and responses to children’s pain

Hierarchical linear regression analyses tested relations among parent mental health symptoms and responses to children’s pain. Separate regression models were tested for protectiveness and catastrophizing. Results are presented in Table 2. Given significant differences in age, education, and income between parents with and without chronic pain, these variables as well as chronic pain status were entered as covariates in all regression models. None of the covariates emerged as a significant predictor in either model.

**Table 2.** Hierarchical linear regression results (*n* = 142).

	Protectiveness				Catastrophizing			
	*B*	SE *B*	β	*P*	*B*	SE *B*	β	*P*
Step 1	*R*^2^ (140) = 0.024				*R*^2^ (140) = 0.098			
Group	2.15	1.73	0.11	0.216	4.30	1.25	0.30	0.001
Parent age	−0.03	0.12	−0.03	0.779	−0.05	0.09	−0.05	0.560
Parent education	−0.06	0.68	−0.01	0.934	−0.01	0.49	0.00	0.997
Parent income	−0.64	0.54	−0.11	0.237	−0.01	0.39	−0.01	0.990
Step 2	*R*^2^ (134) = 0.10, F change = 3.14, *P* = 0.03				R^2^ (134) = 0.24, F change = 8.19, *P* = 0.00			
Depression	0.83	0.32	0.42	0.010	0.70	0.22	0.48	0.002
Anxiety	−0.47	0.39	−0.21	0.227	0.04	0.27	0.02	0.878
Somatization	0.06	0.31	0.03	0.852	−0.18	0.21	−0.12	0.402

As hypothesized, depressive symptoms (β = 0.48, *P* < 0.01) predicted protectiveness in model 1. Specifically, parents who endorsed higher depressive symptoms reported engaging in more protective behaviors. Similarly, as hypothesized, depressive symptoms (β = 0.48, *P* < 0.01) predicted catastrophizing in model 2. Specifically, parents who endorsed higher depressive symptoms also reported engaging in greater catastrophizing about their child’s pain. Contrary to hypotheses, neither anxiety nor somatic symptoms predicted parent protectiveness or catastrophizing about their child’s pain in either model.

### Parent chronic pain moderates the relation between parent anxiety and catastrophizing

**Figure 1.**
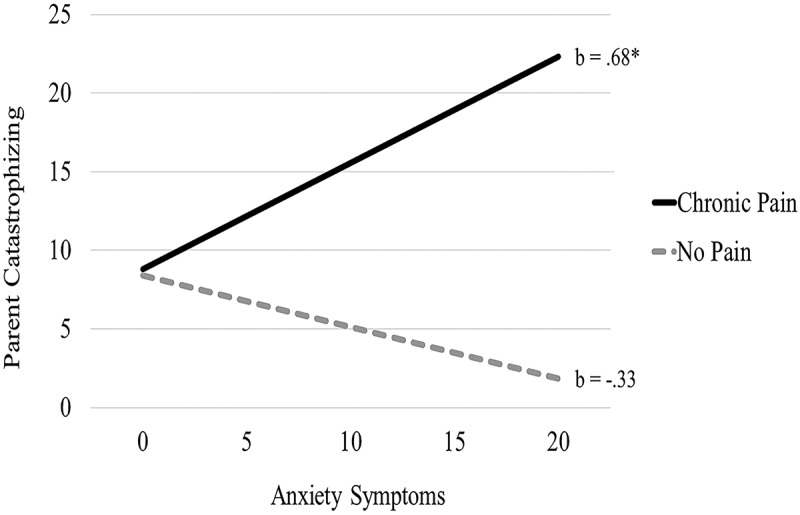
Chronic pain moderates the association between anxiety symptoms and parent catastrophizing about children’s pain.

Moderation analyses were tested using the PROCESS macro in SPSS.^32,33^ This macro uses ordinary least squares regression analyses to simultaneously estimate direct and interaction effects. PROCESS results yield bootstrap estimates that allow for the calculation of an asymmetrical 95% confidence interval. Analyses tested the moderating effect of chronic pain on the relation between parent mental health (i.e., depression, anxiety, somatization) and responses to children’s pain (i.e., protectiveness, catastrophizing). Separate analyses were tested for each mental health symptom (i.e., depression, anxiety, somatization) predicting protectiveness and each mental health symptom predicting catastrophizing. To correct for multiple tests predicting each outcome in three separate moderation analyses, a Bonferroni correction was used, resulting in an adjusted significance level of *P* = 0.0167. Chronic pain moderated the relation between parent anxiety and catastrophizing about children’s pain (β = 5.06, *t *= 2.74, *P *< 0.01), with greater symptoms of anxiety associated with increased catastrophizing about children’s pain in parents with chronic pain only (see Figure 1). No other interaction effect significantly predicted catastrophizing (Depression × Pain status: *t *= 0.27, *P *= 0.79; Somatization × Pain status: *t *= 0.90, *P *= 0.37) or protectiveness (Depression × Pain status: *t* = −0.67, *P* = 0.51; Anxiety × Pain status: *t* = 1.68, *P* = 0.10; Somatization × Pain status: *t* = −0.50, *P* = 0.62).

## Discussion

The goal of the current study was to test parent mental health symptoms (i.e., depression, anxiety, somatization) as predictors of problematic pain-specific parenting responses. Results provide support for the hypothesis that parent mental health symptoms, particularly depression, contribute to protectiveness and catastrophizing responses, above and beyond the presence of parent chronic pain. Additionally, results suggest that parent anxiety may make additional contributions to parent catastrophizing, particularly in parents with chronic pain.

Our results are consistent with previous literature demonstrating that parents with chronic pain experience a range of parenting difficulties.^34^ Parents with chronic pain endorsed significantly higher rates of catastrophizing resulting in a moderate effect size (Cohen’s *d* = 0.64). Protectiveness was similarly higher among parents with chronic pain but only at the trend level (Cohen’s *d* = 0.31). Collectively, these findings suggest that protective responses (e.g., allowing child to miss school) may be common among parents. Parents may benefit from learning strategies to reduce protective and catastrophizing responses to their child’s pain.

Findings from the current study are consistent with the cognitive model of depression^35^ such that parents’ attitudes and cognitions predicted behavior (e.g., responding to children’s distress with greater catastrophizing or protective behavior). Importantly, the relation between parent mental health symptoms and pain-specific parenting responses is likely bidirectional and influenced by other biological and psychosocial variables (e.g., genetic vulnerability, adolescent temperament, adolescent health). Future work is needed to test these relations in the context of a longitudinal study design.

Contrary to hypotheses, neither anxiety nor somatic symptoms contributed uniquely to pain-specific parenting. Notably, at the bivariate level, mental health symptoms (i.e., depression, anxiety, somatization) were each significantly associated with protectiveness and catastrophizing. When included in hierarchical regression models, anxiety symptoms may have dropped to nonsignificant given the substantial overlap between anxiety and depression (*r* = 0.85, 72% shared variance). Regarding somatic symptoms, given the large difference in somatic symptoms by group (i.e., chronic pain versus no chronic pain), it is possible that the variance in somatic symptoms was largely accounted for by group membership. Thus, the presence of higher somatic symptoms may not have made additional contributions. Measuring and interpreting elevations in somatic symptoms in individuals with a chronic illness, particularly chronic pain, is challenging given that pain is commonly associated with additional physical symptoms such as body weakness or faintness, two areas specifically assessed on the somatization subscale of the BSI-18. Moreover, although several important demographic variables were statistically different between parents with and without chronic pain (e.g., income, education), these factors were not associated with protectiveness or catastrophizing and did not contribute uniquely to pain-specific parenting. In addition to testing mental health symptoms as predictors of pain-specific parenting, work is needed to determine sociodemographic variables that may increase environmental risk for child pain.

Moderation analyses found that parents with elevated symptoms of anxiety and chronic pain engaged in greater catastrophizing in response to their child’s pain relative to parents without chronic pain, suggesting that parents with comorbid anxiety and chronic pain are at additional risk for engaging in catastrophizing behavior. Findings further support the importance of evaluating mental health symptoms in chronic pain populations, providing mental health treatment targeting symptom management, and teaching appropriate parenting strategies to respond to their children’s pain. These efforts may help interrupt the intergenerational transmission of chronic pain. Identifying targets for intervention is essential given research demonstrating that children of parents with chronic pain are at increased risk for adverse mental and physical health outcomes.^36^ Importantly, many parents with chronic pain continue to parent quite well, despite the significant challenges and complexities of managing a chronic condition.^37^

The current study has several notable strengths, including use of a clinical sample of biological parents with heterogeneous chronic pain conditions as well as a healthy control group and a focus on early adolescence, a developmental period during which pain in youth increases.^38^ Moreover, this is the first investigation to assess predictors of pain-specific parenting responses and test unique contributions of parent mental health. Importantly, a large subset of participants in the chronic pain group experienced fibromyalgia (40.3%), a chronic pain condition associated with widespread musculoskeletal pain and fatigue. Individuals with fibromyalgia commonly experience impact on mood and cognition^39^; thus, parents in the current sample may be particularly complex, both physically and psychologically. Results of this study should be interpreted in light of limitations. First, cross-sectional data prevent interpretation of causality. Second, our sample primarily included Caucasian biological mothers, which limits generalizability of the findings. Findings may differ among fathers with and without chronic pain. Additional work is needed to recruit a more ethnically diverse sample and test gender differences among caregivers with chronic pain. Moreover, the current study tested parent protectiveness and catastrophizing about pain, though additional pain-specific parenting factors (e.g., attentiveness to pain, discouraging activity) may increase risk for the development of chronic pain in offspring.^11^ Finally, data were derived solely from parent completed self-report measures, which could contribute to common-method bias.

Future research is needed to assess congruence between parental and youth perceptions of parenting behavior in the context of parent chronic pain. Additional work might also test bidirectional and longitudinal models in considering the relations among parent mental health, adolescent mental health, adolescent temperament, and pain-specific parent responses. Daily or momentary assessment of parent mood, anxiety, and pain along with daily or momentary assessment of cognitive and behavioral responses to children’s pain might also shed additional light on the nature of these relationships.
